# Perinatal outcomes and congenital heart defect prognosis in 53313 non-selected perinatal infants

**DOI:** 10.1371/journal.pone.0177229

**Published:** 2017-06-07

**Authors:** Donghua Xie, Hua Wang, Zhiyu Liu, Junqun Fang, Tubao Yang, Shujin Zhou, Aihua Wang, Jiabi Qin, Lili Xiong

**Affiliations:** 1 Department of Information Management, Maternal and Children’s Hospital of Hunan Province, Changsha, Hunan, China; 2 Department of Epidemiology and Health Statistics, School of Public Health, Central South University, Changsha, Hunan, P.R. of China; 3 Department of Health Care Management, Maternal and Children’s Hospital of Hunan Province, Changsha, Hunan, China; 4 Department of Health Care Management, Maternal and Children’s Hospital of Liuyang City, Hunan, China; Institut national de la recherche scientifique, CANADA

## Abstract

**Objective:**

To evaluate perinatal outcomes and congenital heart defect (CHD) prognosis in a non-selected population.

**Methods:**

The population-based surveillance data used in this assessment of CHDs were based on birth defect surveillance data collected from 2010–2012 in Liuyang City, China. Infants living with CHDs were followed up for 5 years to determine their prognosis. Prevalence, prenatal diagnosis, perinatal outcomes, and total and type-specific prognosis data were assessed using SPSS 18.0.

**Results:**

In total, 190 CHD cases were identified among the 53313 included perinatal infants (PIs), indicating a CHD prevalence of 35.64 per 10000 PIs in this non-selected population. The five most frequently identified types of CHDs were ventricular septal defects (VSDs, 38.95%), atrial septal defects (ASDs, 15.79%), cardiomegaly (7.89%), tetralogy of Fallot (TOF, 5.79%), and atrioventricular septal defects (AVSDs, 5.26%). Of the 190 CHD cases, 110 (57.89%) were diagnosed prenatally, 30 (15.79%) were diagnosed with associated malformations, and 69 (36.32%) resulted in termination of pregnancy (TOP). Moreover, 15 (7.89%) PIs died within 7 days after delivery, and 42 (22.10%) died within 1 year. In contrast, 79 (41.58%) were still alive after 5 years. When TOP cases were included, the 5-year survival rate of PIs with prenatally detected CHDs was lower than that of PIs with postnatally detected CHDs (25.45% vs. 63.75%). The CHD subtype associated with the highest rate of infant (less than 1 year old) mortality was transposition of the great arteries (100%). The subtypes associated with higher 5-year survival rates were patent ductus arteriosus (80%), ASD (63.33%), VSD (52.70%) and AVSD (50%).

**Conclusions:**

The rates of prenatal CHD detection and TOP were high in this study population, and the 5-year survival rate of PIs with CHDs was low. The government should strengthen efforts to educate pediatricians regarding this issue and provide financial assistance to improve the prognosis of infants living with CHDs, especially during the first year of life.

## Introduction

Congenital heart defects (CHDs), the most common type of birth defects (BDs), have been reported to occur in 4–8 of every 1000 live births[1, 2] and account for 30–50% of BD-induced infant mortality[3, 4]. Worldwide, epidemiological studies of CHDs have mainly (80%) relied upon population-based surveillance data; however, in China, these studies have predominantly relied upon hospital-based surveillance data[5, 6]. Despite the increased feasibility of this type of surveillance, hospital-based surveillance suffers from the inevitable selection bias associated with the location of delivery and is also affected by geographic location, socioeconomic level and educational status[7–9]. Thus, hospital-based surveillance is potentially associated with increased CHD detection rates compared with population-based surveillance. Therefore, to counteract the disadvantages of hospital-based surveillance, the Chinese Ministry of Health carried out population-based surveillance to assess BDs in 64 counties in 30 provinces (including Liuyang City) in 2006.

Ultrasonography has revolutionized prenatal care and has permitted the detection of a number of major fetal malformations prior to birth[8, 10]. Through the use of this technology, most complicated CHDs can be detected prenatally, increasing the feasibility of prenatal medical and interventional management and helping to guarantee safe delivery at a tertiary center[11]. Additionally, when a fetus is prenatally diagnosed with a severe CHD, elective termination of pregnancy (TOP) can serve as an option for mothers and doctors in many countries, including China[6, 12]. Thus, determination of the perinatal outcomes and the prognosis associated with CHDs may provide important evidence that can inform the provision of medical care.

In recent years, the prevalence of CHDs in Hunan has been the highest in China13]. In this context, the purpose of the present study was to evaluate perinatal outcomes and CHD prognosis in a non-selected population. Briefly, perinatal CHD surveillance data collected from 2010–2012 in Liuyang and 5-year follow-up data were analyzed to better inform antenatal consultations and postpartum treatment.

## Methods

### Study population

This study included all perinatal infants (PIs, including stillbirths, cases of fetal death and live births) and pregnant women residing in Liuyang from 2010–2012 (defined as those registered in the local census register or registered in the nonlocal census register as residing in Liuyang for than 1 year); thus, the study subjects constituted a non-selected population. All records for infants with CHDs were anonymized and deidentified prior to analysis. After we verbally informed the study subjects of the rationale for and the objectives of this study, first, to facilitate data management, we collected basic and important information. We paid greater attention to abnormal cases, including by performing longitudinal follow-up and facilitating their treatment and recovery. Second, according to governmental requirements, we collected and analyzed these data to inform the creation of policies designed to improve the prognosis of infants living with CHDs. Our study was observational in nature, mainly involving monitoring and follow-up that would not disturb the participants. Therefore, the study and the consent procedure were approved by the Medical Ethics Committee of Maternal and Children’s Hospital of Hunan.

### Surveillance

Village doctors made postpartum family visits within 7 days of birth, regardless of whether delivery occurred in the hospital or outside the hospital (3 cases in 2010, 2 cases in 2011, and 5 cases in 2012), according to the recommendations of the *Population-Based Surveillance Scheme of Birth Defects* published by the China National Health and Family Planning Commission (NHFPC). The NHFPC requires all infants to be evaluated for abnormalities by hospital pediatricians. The infants delivered at home were evaluated by midwives and village doctors after delivery. If a newborn was still in the hospital after 7 days, the visit time was delayed accordingly. The attending medical professionals collected basic data for every birth and for infants with a suspected CHD, including birth and family status, as assessed by clinical staff and recorded in the antenatal examination register. The corresponding township hospital confirmed these basic data and filled in infant follow-up data both on paper and online. The township hospital and the county-level maternal and children’s hospital then confirmed the CHD cases using the local hospital information system and filled in the relevant information both on paper and online; this information included birth status, basic family information, outcomes during the surveillance period, and CHD characteristics. The maternal and children’s hospitals and health administrative departments audited the infant follow-up and CHD data, respectively. The monitoring hospitals were inspected and examined at quarterly intervals by county-level administrators and at half-yearly intervals by city-level or province-level administrators to ensure quality control and reduce the probability of misreporting or failure of reporting.

### Follow-up data

The NHFPC’s *National* *Basic* *Public* *Health* *Service* *Specification* *2015* requires that village doctors follow up babies at 28 days; 6 and 12 months; and 1.5, 2, 3, 4, and 5 years and record the babies’ medical condition, in addition to performing a physical examination, providing treatment and determining survival. Using this resource, we collected data on the survival and treatment of infants living with CHDs who were under the age of 5 years[S1 File]. Additionally, we verified the main data recorded therein via telephone.

### Criteria for CHD diagnosis

The doctors diagnosed CHDs and data management staff confirmed CHD diagnoses according to the definitions provided in the *Maternal and Children’s Health Monitoring Manual in China* (MMCHMM) and the *Chinese National Criteria for Birth Defects and Tiny Deformities* (CNCBDTD), which have been published by the NHFPC (postural defects are excluded from the monitoring system). The main method used for CHD diagnosis was four-chamber echocardiography. All CHD cases were confirmed by experts after delivery. Clinical diagnosis of CHDs was established within 42 days after delivery. Only cases with isolated patent foramen ovale (PFO) or patent ductus arteriosus (PDA) ≥3 mm were included in this study.

### Statistical analysis

Gender-specific CHD prevalence rates and their corresponding 95% confidence intervals (CIs) were calculated. The rates of CHDs and other BDs, population characteristics, subtypes of CHDs and associated malformations were also assessed. Mortality rates were calculated for both the prenatally detected group and postnatally detected group and compared using the Chi-squared test. Prevalence estimates are reported per 10000 PIs. The data were analyzed using SPSS 18.0, and statistical significance was set at P<0.05.

## Results

### Population

The study population included 53313 PIs, of which 190 had been diagnosed with CHDs, for a total prevalence of 35.64 per 10000 PIs (95% CI: 34.12–37.26). The prevalence rates identified in male and female infants were 36.23 per 10000 PIs (103/28426) (95% CI: 34.11–38.45) and 34.96 per 10000 PIs (87/24885) (95% CI: 34.37–35.55), respectively. Of the assessed infants, 16.84% (32/194) had been diagnosed with CHDs or other BDs. The median maternal ages were 25.74 (15–48) years in the overall population and 25.33 (16–41) years in the mothers of infants with CHDs. The median gestational age was 38.99 (28–49) weeks for the overall population and 35.80 (28–41) weeks for the infants with CHD.

### Spectrum of CHDs and associated malformations

**Fig 1:**
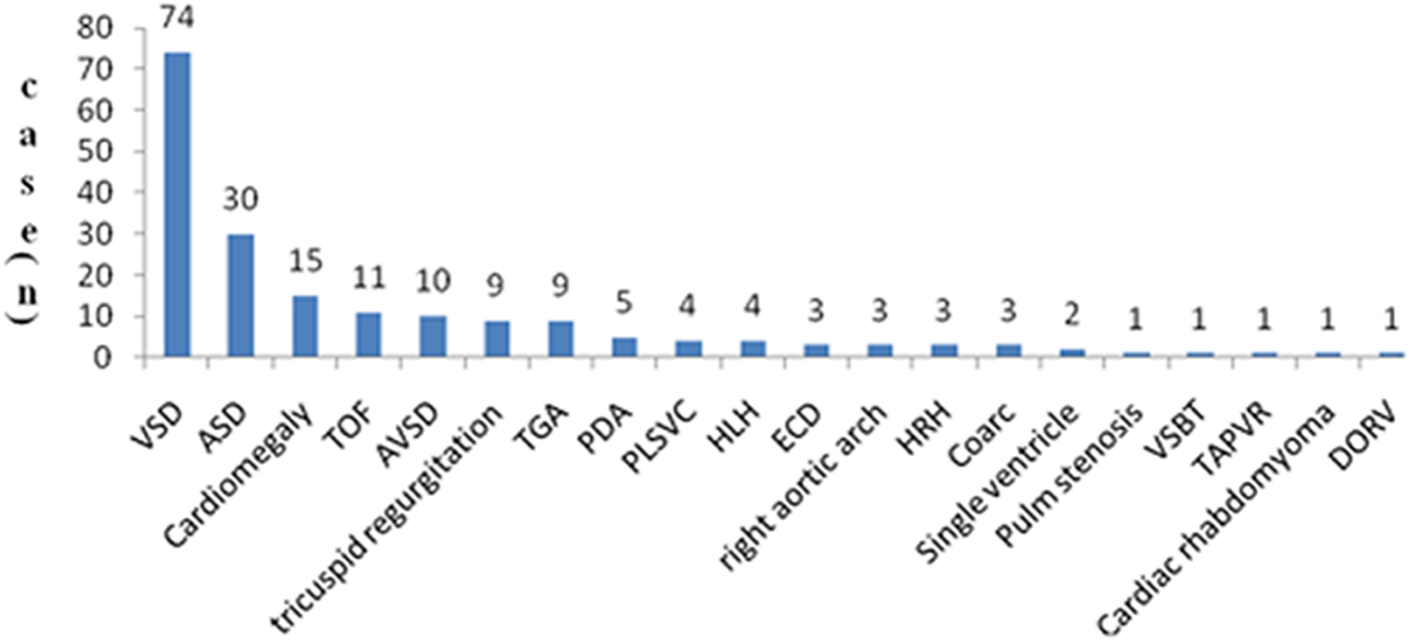
Spectrum of CHDs in a non-selected population of 53313 PIs. The most severe types of CHDs were defined as those in which several types of CHDs occurred simultaneously. VSD: ventricular septal defect; ASD: atrial septal defect; TOF: tetralogy of Fallot; AVSD: atrioventricular septal defect; TGA: transposition of the great arteries; PDA: patent ductus arteriosus; PLSVC: persistent left superior vena cava; HLHS: hypoplastic left heart; ECD: endocardial cushion defect; HRHS: hypoplastic right heart; Coarc: coarctation of the aorta; VSBT: ventricular septal bulging tumor; TAPVR: total anomalous pulmonary venous return; DORV: double-outlet right ventricle; cardiomegaly: major characteristics of cardiomegaly without specific diagnosis.

The five most frequently identified types of CHDs were ventricular septal defects (VSDs) (38.95%, 74/190), atrial septal defects (ASDs) (15.79%, 30/ 190), cardiomegaly (7.89%, 15/190), tetralogy of Fallot (TOF) (5.79%, 11/190), and atrioventricular septal defects (AVSDs) (5.26%, 10/190) (Fig 1).

**Fig 2:**
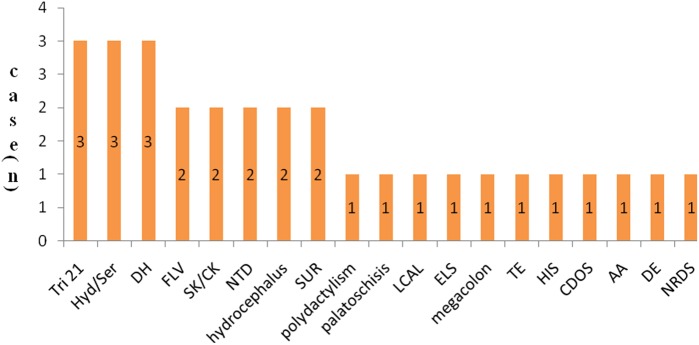
Spectrum of associated malformations in a non-selected population of 53313 PIs. Tri 21: trisomy 21 syndrome; SUR: single umbilical artery; Hyd/Ser: hydrothorax/seroperitoneum; DH: diaphragmatic hernia; FLV: fetal lateral ventriculomegaly; SK/CK: single kidney/compound kidney; NTD: neural tube defect; LCAL: lung cystic adenomatoid lesion; ELS: enlargement of the liver and spleen; TE: talipes equinovarus; HIS: internal heterogeneous syndrome; CDOS: congenital dysplasia of the skin; AA: accessory auricle; DE: diaphragmatic eventration; NRDS: neonatal respiratory distress syndrome.

Fig 2 shows the spectrum of associated malformations, with the most frequently identified types being Tri 21 (10%), SUR (10%), Hyd/Ser (10%), and DH (10%).

### Outcomes and prognosis of patients with CHDs detected at different times

**Fig 3:**
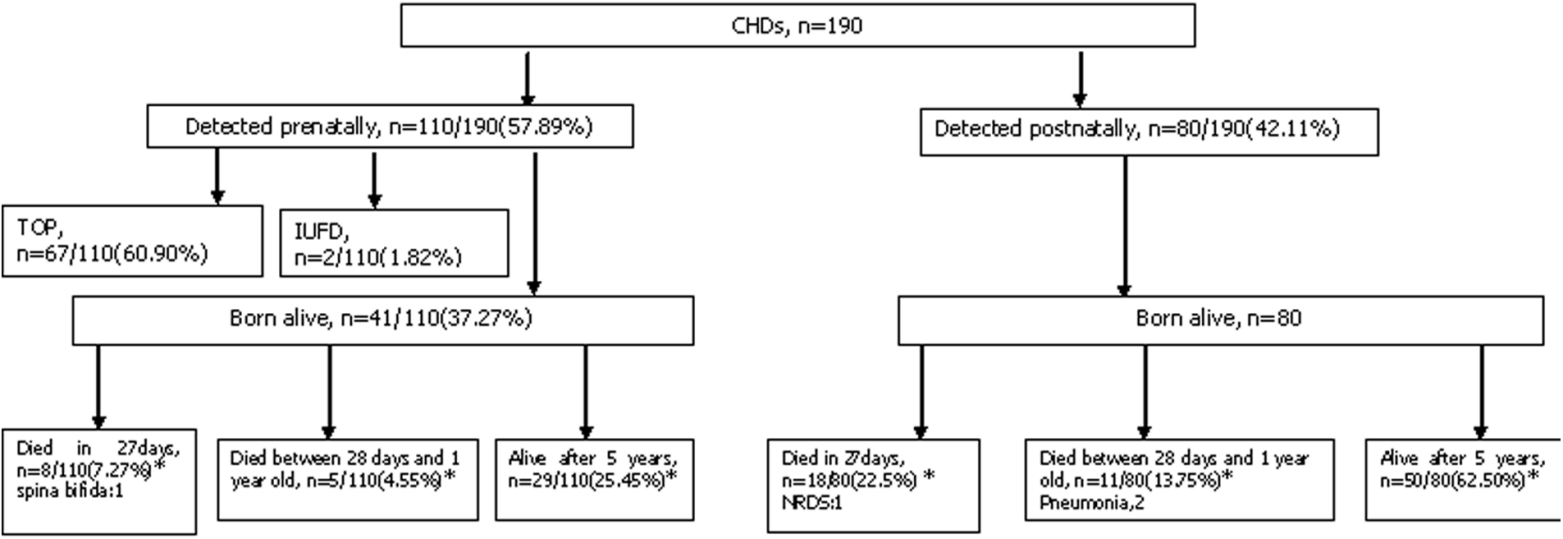
Outcomes and prognosis of infants with CHDs detected either prenatally or postnatally. *P*<*0.001.

In the 190 identified PIs, CHDs with were detected either prenatally (110, 57.89%) or postnatally (80, 42.11%). In total, 67 (60.90%) cases in the prenatally detected group ended in TOP, and 2 (1.82%) intrauterine fetal deaths (IUFDs) and 41 (37.27%) live births were identified. Including the TOP cases, the 27-day mortality rate, medium-term mortality rate (the mortality rate between 28 days and 1 year of life), and 5-year survival rate observed in the prenatally detected group were all significantly lower than those in the postnatally detected group: 7.27%, 8/110 vs. 22.5%, 18/80; 4.55%, 5/110 vs. 13.75%, 11/80; and 25.45%, 29/110 vs. 63.75%, 50/80, respectively (P<0.001) (Fig 3). If the terminated pregnancies were excluded, no significant between-group differences in 5-year survival rates (68.29% vs. 63.75%), 27-day mortality rates (19.51% vs. 22.5%), or medium-term mortality rates (12.20% vs. 13.75%) were observed. These findings suggested that prenatal diagnosis and prenatal counseling played significant roles in the determination of the outcomes of infants living with CHDs in Hunan.

### Outcome and prognosis of subtypes of CHDs

As shown in Table 1, because of prenatal diagnosis of CHDs, the rates of TOP were 36.32% (69/190) among PIs with CHDs overall (including 2 IUFDs) and 62.72% (69/110) among PIs prenatally diagnosed with CHDs. The time from delivery to death was most frequently 1 year or less (22.10%, 42/190), and deaths occurred especially frequently within 7 days (7.89%, 15/190). Of the 190 PIs with CHDs, 20 (10.53%) underwent surgery, 9 (4.73%) were waiting to undergo surgery, 10 (5.26%) did not undergo surgery for different reasons (e.g., economic or medical reasons), and 79 (41.58%) were alive after 5 years. The subtypes associated with higher rates of infant (less than 1 year old) mortality included TGA (100%), HLHS (100%), and HRHS (100%). The subtypes associated with 5-year survival rates greater than 50% were PDA (80%), ASDs (63.33%), VSDs (52.70%) and AVSDs (50%).

**Table 1:** Clinical management and follow-up of 190 CHD cases.

CHD subtype			Associated malformation		Prenatal diagnosis		TOP (including IUFD)		Death within 7 days		Death between 7 days and 27 days		Death between 28 days and 1 year		Total deaths occurring under the age of 1 year		Surgery			Alive >5 years	
	n	%	n	%	n	%	n	%	n	%	n	%	n	%	N	%	Completed	Upcoming	None	n	%
VSD	74	38.95	8	10.81	45	60.81	22	29.73	2	2.70	5	6.76	6	8.11	13	17.57	11	5	7	39	52.70
ASD	30	15.79	5	16.67	6	20	4	13.33	4	13.33			3	10.00	7	23.33	3	1		19	63.33
Cardiomegaly	15	7.89	5	33.33	14	93.33	10	66.67	2	13.33	1	6.67	0	0.00	3	20.00	1	1		2	13.33
TOF	11	5.79			7	63.64	7	63.64	1	9.09			1	9.09	2	18.18	2			2	18.18
AVSD	10	5.26	3	30	3	30	2	20	1	10.00	2	20.00	0	0.00	3	30.00	1		1	5	50.00
Tricuspid regurgitation	9	4.74	3	33.33	7	77.78	5	55.56			1	11.11	0	0.00	1	11.11		1		3	33.33
TGA	9	4.74			5	55.56	4	44.44	1	11.11	1	11.11	3	33.33	5	55.56			1	0	0.00
PDA	5	2.63			0	0	0	0					1	20.00	1	20.00		1		4	80.00
PLSVC	4	2.11			3	75	2	50					1	25.00	1	25.00			1	1	25.00
HLHS	4	2.11	1	25	3	75	1	25	3	75.00			0	0.00	3	75.00				0	0.00
ECD	3	1.58	1	33.33	3	100	1	33.33					1	33.33	1	33.33	1			1	33.33
Right aortic arch	3	1.58			3	100	2	66.67					0	0.00	0	0.00				1	33.33
HRHS	3	1.58	2	66.67	3	100	3	100					0	0.00	0	0.00				0	0.00
Coarc	3	1.58	1	33.33	3	100	1	33.33	1	33.33			0	0.00	1	33.33				1	33.33
Single ventricle	2	1.05	1	50	2	100	2	100					0	0.00	0	0.00				0	0.00
Pulmonary stenosis	1	0.53			0	0	0	0					0	0.00	0	0.00	1			1	100.00
Ventricular septal bulging tumor	1	0.53			1	100	1	100					0	0.00	0	0.00				0	0.00
TAPVR	1	0.53			0	0	0	0			1	100.00	0	0.00	1	100.00				0	0.00
Cardiac rhabdomyoma	1	0.53			1	100	1	100					0	0.00	0	0.00				0	0.00
DORV	1	0.53			1	100	1	100					0	0.00	0	0.00				0	0.00
Total	190	100.00	30	15.79	110	57.89	69	36.32	15	7.89	11	5.79	16	8.42	42	22.11	20	9	10	79	41.58

## Discussion

The prevalence of CHDs in the evaluated population was lower than that previously identified in hospitals (35.64 vs. 53.1 per 10000 PIs)[13] in Hunan. The likely reasons for this difference were described in the introduction section. The prevalence of CHDs identified in our research was similar to that previously reported in other rural areas in China[14], and the total prevalence of CHDs was lower than other population-based estimates in other countries[10]. The reason for these differences may be that population-based surveillance in China is carried out from 28 gestational weeks to 42 days after delivery, whereas surveillance is carried out from 20 gestational weeks to 1 or 2 years or even longer in other countries, such as the USA, Australia and Finland[15]. As previously reported, the mean gestational age at diagnosis is 26 weeks[16, 17], which means that certain severe CHD cases diagnosed before 28 gestational weeks (which were TOP cases) were excluded from our study. In addition, the prevalence of CHDs varies by race, socioeconomic status and geographic location[18].

Several studies have addressed prenatal rates of CHD detection[9, 19, 20]. However, comparing our results with those of other studies might be difficult due to differences in population selection, study design and CHD classification. The results of our study showed that the overall prenatal rate of CHD detection in PIs was 57.89%, which was consistent with the finding of another study (57%)[10].

The rate of TOP identified for PIs with CHDs indicates the potential impact of prenatal diagnosis, which plays a role in the prevention of live births with CHDs[21]. As previously reported, the rate of TOP due to the presence of abnormalities varies significantly across countries[12]. The rate of TOP in PIs identified as having CHDs prior to 24 gestational weeks was 36% in Bold’s research[22] and 53% in a study by Tegnander; these rates were lower than our results, detailing the rate of TOP at more than 28 gestational weeks (60.90%). This discrepancy may be attributable to differences in prenatal diagnosis techniques, laws and ethics beliefs related to TOP[23]. As reported, once a severe CHD is diagnosed prenatally, the TOP rate is more than 90% in China, which means that TOP due to CHDs is common in China[24].

The 5-year survival rate (41.58% among the PIs with CHDs overall, 86.81% among infants living with CHDs) in our study was lower than that reported by certain other studies[10, 25]. As described earlier, of the 97 PIs with major CHDs, 49 (50.52%) survived past the age of 2. Of the 321 fetuses with minor CHDs, 310 (97%) were alive after 2–13 years of follow-up. This phenomenon may be attributable to several factors. First, data suggest that there are 0.14 pediatricians per 1000 children in Liuyang, which is lower than the rate observed in China overall (0.53 pediatricians per 1000 children) and the rates reported in developed countries (0.85–1.3 pediatricians per 1000 children)[26]. Worse, the educational degrees and professional titles of pediatricians in Liuyang City have been found to be lower in level than those reported elsewhere[27]. Second, Liuyang has been classified as an economically undeveloped rural area. Research conducted by Yingyao Chen of FuDan University showed that each CHD case is associated with an average cost of $14541.65[28]. In our study, 10 CHD infants did not undergo surgery due to economic reasons or disease severity (with 5 deaths), which may have resulted in a reduced 5-year survival rate. In addition, after a 1-year follow-up period, no more deaths were identified among the infants living with CHDs who survived until 5 years old, indicating that more attention should be paid during the first year of life to improve the survival of CHD patients. Interestingly, despite similar rates of prenatal detection, the rate of TOP among PIs with CHDs and the five-year survival rate among infants living with CHDs were found to be higher in Liuyang City than in Guangdong Province[24] (Table 2). This phenomenon may be interpreted as follows. First, 99.34% of mothers received prenatal care in Liuyang from 2010–2012, and complicated CHDs need to be assessed at a provincial prenatal diagnosis center. Second, the average GDP in Liuyang City in 2015 was $5220.30[29], which was lower than that identified in Guangdong Province in 2015 ($9843.20)[30]. Third, the number of health technician personnel per 10000 population in Liuyang City was 50, which is lower than that in Guangdong Province (57)[30].

**Table 2:** Comparison of outcomes and prognosis of infants with CHDs between Liuyang City and Guangdong Province.

Area	CHDs (n)	Rate of prenatal detection	Rate of TOP among detected cases*	Five-year survival rate among infants living with CHDs*
Liuyang City	190	57.89% (110/190)	62.73% (69/110)	65.29% (79/121)
Guangdong Province	151	64.90% (98/151)	47.96% (47/98)	91.35% (95/104)

*P<0.05

The present study included data from a large non-selected population. The advantages of our study were that data collection was initiated at 28 gestational weeks and that cases were followed up for a period of 5 years. However, there were certain limitations. First, we had no information regarding TOP at less than 28 gestational weeks among mothers of infants with severe CHDs, which could have led to underestimation of the total prevalence of CHDs. Second, the rates of chromosome examination and autopsy were low, which is a common problem in China; this problem could have reduced the rates of detection of CHDs and their associated malformations, and especially chromosomal disease. Third, certain asymptomatic CHDs may have remained unidentified if an infant was born to a mother who did not receive prenatal care at the appropriate times; however, these cases were likely few in number (the rate of prenatal care in Liuyang was 99.34%). Fourth, several months have elapsed between the end of the 5-year follow-up of 2012 births and the present, which may have resulted in a lack of identification of certain (but likely few) deaths. Lastly, the numbers of cases of certain subtypes of CHDs were limited, especially for TAPVR (1 case), DORV (1 case), and cardiac rhabdomyoma (1 case), and, thus, the data may not completely represent the outcomes of affected patients.

In sum, the rate of prenatal CHD detection and the rate of TOP were high within our study population, and the 5-year survival rate of PIs with CHDs was very low. The government should strengthen efforts to educate pediatricians regarding this issue and provide financial assistance to improve the prognosis of infants living with CHDs, especially during the first year of life.

## Supporting information

S1 File Follow-up data of 190 CHD PIs 20170518. (XLSX)
